# Stakeholders’ contributions to tailored implementation programs: an observational study of group interview methods

**DOI:** 10.1186/s13012-014-0185-x

**Published:** 2014-12-06

**Authors:** Elke Huntink, Jan van Lieshout, Eivind Aakhus, Richard Baker, Signe Flottorp, Maciek Godycki-Cwirko, Cornelia Jäger, Anna Kowalczyk, Joachim Szecsenyi, Michel Wensing

**Affiliations:** Radboud University Medical Center, Radboud Institute for Health Sciences, PO Box 9101, 6500 HB Nijmegen, the Netherlands; Research Centre for Old Age Psychiatry, Innlandet Hospital Trust, 2312 Ottestad, Norway; Norwegian Knowledge Centre for the Health Services, P.O. Box 7004, St. Olavs plass, N-0130 Oslo Norway; University of Leicester, 22-28 Princess Road West, Leicester, LE1 6TP UK; University of Oslo, Postboks 1089 Blindern, 0317 Oslo, Norway; Medical University of Lodz, ul. Kopcinskiego 20, 90-153 Lodz, Poland; Heidelberg University Hospital, Voßstraße 2, D-69115 Heidelberg, Germany; Centre for Family and Community Medicine, Medical University of Lodz, ul. Kopcinskiego 20, 90-153 Lodz, Poland

**Keywords:** Group interview methods, Methodology/methods, Chronic illness care, Implementation science, Evidence-based practice

## Abstract

**Background:**

Tailored strategies to implement evidence-based practice can be generated in several ways. In this study, we explored the usefulness of group interviews for generating these strategies, focused on improving healthcare for patients with chronic diseases.

**Methods:**

Participants included at least four categories of stakeholders (researchers, quality officers, health professionals, and external stakeholders) in five countries. Interviews comprised brainstorming followed by a structured interview and focused on different chronic conditions in each country. We compared the numbers and types of strategies between stakeholder categories and between interview phases. We also determined which strategies were actually used in tailored intervention programs.

**Results:**

In total, 127 individuals participated in 25 group interviews across five countries. Brainstorming generated 8 to 120 strategies per group; structured interviews added 0 to 55 strategies. Healthcare professionals and researchers provided the largest numbers of strategies. The type of strategies for improving healthcare practice did not differ systematically between stakeholder groups in four of the five countries. In three out of five countries, all components of the chosen intervention programs were mentioned by the group of researchers.

**Conclusions:**

Group interviews with different stakeholder categories produced many strategies for tailored implementation of evidence-based practice, of which the content was largely similar across stakeholder categories.

**Electronic supplementary material:**

The online version of this article (doi:10.1186/s13012-014-0185-x) contains supplementary material, which is available to authorized users.

## What is new?

### Key findings

- Group interviews with stakeholders (researchers, quality officers, health professionals, and external stakeholders) provided many strategies for implementing evidence-based chronic illness care.
- Despite differences in numbers of suggested strategies, the type of suggested strategies for implementing evidence-based practice did not systematically differ between the different stakeholder categories.
- The added value of the structured interview after brainstorming was variable, but overall, it provided a substantial number of new suggestions for strategies.

### What this adds to what is known

- Group interviews with healthcare professionals and researchers were most productive in generating strategies for implementing evidence-based practice in healthcare for patients with chronic diseases.
- The different stakeholder categories largely provided similar types of strategies for improving healthcare practice.

### What is the implication, what should change now

- There seemed to be consensus among stakeholders regarding the type of strategies thought likely to be effective in implementing evidence-based chronic illness care.
- Given the scarcity of research on interview methods for tailored implementation, further research on this topic is recommended.

## Background

Tailored implementation strategies intend to target relevant determinants of practice (also called “barriers and facilitators” for change), which is expected to contribute to their effectiveness [1]. This claim is supported by a systematic review of trials of such strategies, which found an overall positive effect of tailored implementation [2]. However, a qualitative analysis of the methods used for tailoring found substantial heterogeneity and little indication of the usefulness of any method [3]. Comparative studies are needed of different methods for generating strategies for improving healthcare practice. Here, we focus on the potential value of group interviews with different stakeholder groups with this purpose, most particularly on brainstorming followed by structured group interviews [4,5].

A previous study provided a content analysis of the types of strategies for evidence-based practice mentioned by different stakeholders [6], using a previously developed framework [7]. In the present study, we assessed the usefulness of group interviews with stakeholders in terms of numbers and use of suggestions and the added value of different stakeholder groups and interview techniques. Group interviews were chosen because these were perceived by the research team as potentially valid and feasible methods for generating ideas. The main objectives of the study were (a) to compare the number and types of strategies generated by different stakeholders in brainstorm sessions, (b) to assess the added value of a structured group interview after brainstorming, and (c) to assess whether stakeholders provided strategies that were actually included in tailored intervention programs, which were subsequently tested in cluster randomized trials. Subsequently, we assessed the types of the strategies based on the framework of determinants of practice with seven domains.

## Methods

### Study design

A prospective observational study was conducted in five countries: Germany, the Netherlands, Norway, Poland, and the United Kingdom (UK). Group interviews with relevant stakeholders were done in the autumn of 2012 based on a written study protocol, which was developed by the group of authors (Additional file 1). Ethical committees in the five countries assessed the study protocol and waived or gave approval (Ethics Committee Heidelberg (Germany), Bioethics Committee of the University of Lodz (Poland), Committee for Research in Humans Radboudumc (Netherlands), Regional Committee for Medical and Health Research (Norway), NRES Committee London - Camden & Islington (UK)).

### Setting

This study was part of the Tailored Implementation for Chronic Diseases (TICD) project [8], which aimed to provide insight into the usefulness and effectiveness of methods for tailoring implementation interventions to determinants of practice in chronic illness care. Five different chronic conditions were targeted in five different countries: multi-morbidity (Germany), cardiovascular risk management (CVRM) (the Netherlands), depression in the elderly (Norway), chronic obstructive pulmonary disease (COPD) (Poland), and obesity (UK). In each country, a set of three to six specific evidence-based recommendations were chosen as targets throughout the studies. Subsequently, determinants were identified to enhance those recommendations, using empirical studies guided by a newly developed checklist. In this checklist, 57 potential determinants were defined and grouped in seven domains which are the following: guideline factors, individual health professional factors, patient factors, professional interactions, incentives and recourses, capacity for organizational change, and social, political and legal factors [7]. In this study, we focused on the subsequent phase, which aimed at generating strategies for improving healthcare practice. In the final phase of the TICD project, tailored interventions will be evaluated in cluster randomized trials [9-13].

### Study population

In each country, the study involved at least four different categories of stakeholders. Convenience sampling (using a variety of methods) was used to purposefully recruit by mail or email different categories of individuals into groups. Category 1 consisted of healthcare researchers, including members of the TICD project teams and other healthcare researchers. Category 2 comprised quality improvement officers: individuals who develop or coordinate continuing medical education and quality improvement for the targeted patients, professionals or healthcare sector workers. Category 3 comprised healthcare professionals like primary care physicians and primary care nurses. For category 4 authorities, health insurers or other purchasers of healthcare were invited. Additionally, the country research team could decide to include extra group interviews. A fifth category comprised patients and/or relatives. These were only included in the Netherlands and Norway. Each individual joined only one group and most of the participants did not know each other. Patients gave written informed consent for participation; all other participants consented by actual participation.

### Group interviews

The group interviews followed a standardized procedure, although the content of the questions and responses differed across countries, depending on the clinical condition and the healthcare system. The interviews consisted of a brainstorming phase followed by a structured interview phase; for each phase, 1 h was indicated. A group moderator gave an oral presentation at the start of the brainstorming and at the start of the structured interviews. The moderator, who was experienced in leading a group interview, led the interview and took care that the groups did not focus on study designs, research methods, or outcome measures. An observer (present in some countries) recorded all strategies, made field notes, and added question prompts as needed.

The group sessions started by providing a brief general introduction about the TICD project and information about the specific chronic condition followed by the recommendations targeted for implementation (between three and six per country) and the list of prioritized determinants of practice identified in previous research (between 11 and 33 per country) [14]. Using the principles of brainstorming, participants were then invited to suggest interventions and policies to address the determinants and ways to achieve the targets for improvement. The main rules were that criticism had to be avoided, combination and extension of previously suggested strategies was encouraged, and “wild” strategies were welcomed [15]. There was no limit to the number and type of the strategies. No direction or guidance was given except that major omissions regarding goals were signaled by the moderators. After a short break, a short presentation provided information on implementation strategies and research evidence related to their potential impact in the chosen clinical condition in each country to focus on the gaps with recommended practice. This presentation had been prepared before the session and was the same for all group interviews in a country. This was the introduction to the structured interviews, in which additional targets of improvement and domains of determinants of practice were systematically explored, using open questions. Field notes were made by using structured schedules (relating to the targets of improvement and domains of determinants of practice) to fill in. Interviews were not audio taped.

### Data analysis

In each country, the national research team listed the strategies in a structured document and translated these into English. These data were sent to the Dutch team which transferred them into a standardized data file for further analysis. The different research teams checked and approved the results of the different phases in this research.

We listed the numbers of strategies of the different categories of stakeholders in the two phases of the group interviews (brainstorming versus structured interview). The numbers of strategies were counted per country, group, and interview phase. Crude figures refer to items regardless of how many similar ones were mentioned. One researcher determined how many unique strategies were provided in each interview during the brainstorming phase. Next, the same researcher assessed the number of unique strategies added in the structured phase of the interview compared to the results of the preceding brainstorming phase. This resulted in the numbers of unique strategies per phase, per group, and per country.

One researcher determined how many unique strategies were provided per group, per phase (and how many unique strategies were added in the structured interviews compared to the results of the brainstorming phase), and per country. This was checked independently by a second researcher. Any discrepancies were resolved by consensus. We tended towards listing strategies as unique, unless they were the same or very close to another idea. We also assessed which strategies contributed to the tailored intervention program (including strategies of the groups of patients and patients’ relatives) for each country.

For analysis, we compared the numbers of crude and unique strategies between groups and between interview phases within each country (including strategies of the groups of patients and patients’ relatives). A qualitative content analysis of the items has been reported elsewhere [6]. A multiple linear regression analysis was performed to explore the relationship between the number of strategies mentioned and the time spent on the interview (anticipating that longer interviews would provide more strategies). Likewise, we assessed the relationship between the number of strategies and the number of participants in the group interview (anticipating that groups with more individuals would provide more strategies). For this analysis, the stakeholders interviewed in two groups were analyzed as separate groups. Norwegian interview time was not available, so Norwegian data were not included in this analysis.

Two researchers categorized the strategies gathered during brainstorming as well as new strategies mentioned in the structured phase in relation to the given set of determinants of practice. We assessed the types of the strategies based on the framework of determinants of practice with seven domains [7]. This analysis was performed *post hoc*; a significant difference was set at *p* < 0.01. The chi-square test was used to examine whether the distribution of the types of strategies per country differed systematically between stakeholder groups. The statistical analyses were done in SPSS, version 20.

## Results

### Descriptive data

Overall, 25 group sessions were held in five different countries involving 127 individuals. Groups varied in size from three to nine participants (Table 1), and the group interviews lasted on average 112 min (range 67–135 min). During brainstorming, a total of 881 unique strategies were generated and the structured interviews provided a total of 225 additional unique strategies. Overall, the participants generated a total of 1,106 unique strategies. The differences in the numbers of strategies were larger between countries than between groups within a country (Table 2).

**Table 1:** **Number of participants in the group interviews (** ***n*** **= 127 individuals)**

	**Germany (multi-morbidity)**	**Netherlands (CVRM)**	**Norway (depression by elderly)**	**Poland (COPD)**	**UK (obesity)**	**Totals**
Implementation researchers	5	7	4	4	6	26
Quality improvement officers	7	3	5	3^a^	4	22
Healthcare professionals	4	14^b^ (9 + 5)	11^b^ (5 + 6)	4	9^b^ (4 + 5)	42
Authorities, health insurers, and other purchasers of healthcare	4	5	6	4	6	25
Patients or their relatives	-	12^b^ (4 + 8)	3	-	-	15
Totals	20	41	29	15	25	

^a^Individual interviews; ^b^two groups interviewed.

**Table 2:** **Numbers of strategies provided in brainstorm phases and structured phase**

		**Brainstorm phases**	**Structured phase**			
		**Number of crude and unique strategies** ^**c**^	**Crude number of strategies**	**Unique strategies within this group and phase**	**Additional unique strategies suggested in structured phase compared to brainstorm phase per group (% of all unique strategies in structured phase)**	**Total of unique strategies per group per country**
Healthcare researchers	Germany	38	8	8	7 (88%)	45
	Netherlands	20	28	14	12 (86%)	32
	Norway	35	-	-	-	35
	Poland	18	17	17	0 (0%)	18
	UK	49	16	16	8 (50%)	57
Quality improvement officers	Germany	33	5	5	4 (80%)	37
	Netherlands	19	27	27	27 (100%)	46
	Norway	99	-	-	-	99
	Poland^a^	21	21	21	0 (0%)	21
	UK	22	7	7	7 (100%)	29
Healthcare professionals	Germany	21	12	12	12 (100%)	33
	Netherlands^b^	36	76	55	55 (100%)	91
	Norway^b^	120	-	-	-	12
	Poland	8	8	8	0 (0%)	8
	UK^b^	81	23	23	23 (100%)	104
Authorities, health insurers, and other purchasers of healthcare	Germany	32	9	9	9 (100%)	41
	Netherlands	24	35	22	22 (100%)	46
	Norway	93	-	-	-	93
	Poland	13	14	13	1 (7%)	14
	UK	28	13	13	3 (23%)	31
Patients and relatives of patients	Netherlands^b^	36	42	37	35 (95%)	71
	Norway	35	-	-	-	35
Total		881	361	307	225	1106

Totals in brainstorm per country: Germany *n* = 124, Netherlands *n* = 135, Norway *n* = 382, Poland *n* = 60, UK *n* = 180.

^a^Individual interviews; ^b^two groups interviewed; ^c^crude items equaled unique items in the phase.

In Norway and the UK, interviews with primary care physicians and primary care nurses were held separately. In the Netherlands, a mixed group of primary care physicians and primary care nurses and a group of hospital-based vascular nurses were interviewed. The data of stakeholders interviewed in two groups were merged as one group. The Norwegian team did not include the structured interviews as these were not feasible in their setting. The Polish team held three individual interviews with quality improvement officers for feasibility reasons; these data were merged as one and used when appropriate. Data of patients or their relatives were not used in comparative analyses because only two countries performed these interviews. The number of strategies generated during brainstorming was related to interview time and group size, but only a very low proportion of the variation was explained by these two factors (R-square 0.014 for the brainstorm phase and 0.037 for the structured interview). As their impact was low, all further analyses are uncorrected for interview time and number of participants.

### Comparison of number of strategies between stakeholders

Table 2 facilitates a comparison of the number of strategies between stakeholder groups. Focusing on the crude number of strategies generated during brainstorming, healthcare professionals provided the most strategies in three countries: the Netherlands (*n* = 36, 36% of all strategies in this country), Norway (*n* = 120, 34%), and the UK (*n* = 81, 45%). Healthcare researchers provided the most strategies in Germany (*n* = 38, 31%) and in Poland (*n* = 18, 46%).

### Comparison of types of strategies between stakeholders

The types of strategies from brainstorming did not systematically differ between stakeholder groups within each of the countries, except for the Netherlands (*X*^2^ (15, *n* = 99) = 35.693 *p* = 0.002). In this country, quality improvement officers mentioned more strategies aimed at the individual professional, while the healthcare professionals mentioned more strategies aiming at patient factors. There were no significant differences regarding types of strategies from the structured phase in any of the participating countries. This analysis was performed *post hoc*, and for each country, the results of brainstorming and structured interviews (except Norway) were analyzed separately (a total of nine statistical tests).

### Number of strategies added in structured interviews

For this analysis, we focused on the unique strategies that were identified during brainstorming and the structured interviews (Table 2). Brainstorming generated 8 to 120 unique strategies per group; the structured interviews added 0 to 55 unique strategies. The highest numbers of additional strategies in the structured interviews of all groups together were found in the Netherlands (*n* = 116, 54% of all unique strategies in this country) and the UK (*n* = 41, 19%). In Germany, 32 (21%) unique strategies were added to the unique strategies of the brainstorming. In Poland, only one (2%) additional item was made during the structured interviews.

### Use of strategies in intervention programs

Table 3 describes the tailored intervention programs which were developed based on the results of this research and will be evaluated in cluster randomized trials. This analysis also included strategies identified by the individual interviews in Poland and the group interviews with patients in the Netherlands and relatives of patients in Norway (Table 4). In each country, all groups mentioned strategies which contributed to the tailored intervention programs. Strategies which were incorporated in the intervention programs were mostly mentioned during brainstorming, except in the Netherlands.

**Table 3:** **The tailored intervention program for each European country**

Germany	1.Training on polypharmacy of primary care clinicians
	2. Development and sharing of practice concepts (local protocols)
	3. Provision of checklist for medication counseling and medication review
	4. Provision of template for medication list
	5. Provision of tablet PC with self-learning program
	6. Campaign with posters and leaflets
Netherlands	1. Refresher motivational interviewing training for primary care nurses
	2. E-learning module on cardiovascular risk management for primary care nurses
	3. Local treatment protocol for cardiovascular patients.
	4. Card with treatment values
	5. Support and encouragement of primary care nurses to use e-health applications for patients without symptoms of depression
	6. Support and encouragement of primary care nurses to refer patients with mild symptoms of depression to physical activity groups
	7. Support and encouragement of primary care nurses to refer patients with severe symptoms of depression to depression treatment
Norway	1. Tools and checklist for developing collaborative care plans for municipalities
	2. Information resources for healthcare professionals on treatment options
	3. Information resources for patients and relatives
	4. Educational outreach visits to primary care practices.
	5. E-learning resources, including CME courses
	6. Comprehensive website with information and educational resources.
Poland	1. Training on stop-smoking counseling in primary care physicians.
	2. Dyspnoe scale attached to patient records
	3. Checklist for managing COPD patients
	4. Provision of training inhaler devices to practices.
United Kingdom	1. Training and scripts for counseling patients for primary care clinicians
	2. Training in waist measurement for primary care clinicians
	3. Educational booklets for patients
	4. Discussion on revision of roles regarding obese patients in practices
	5. Provision of information on local pathways

**Table 4:** **Number of strategies used for the intervention programs**

	**Countries (number of parts in the intervention program)**				
	**Germany (6)**	**Netherlands (7)**	**Norway (6)**	**Poland (4)**	**UK (5)**
Healthcare researchers	6	5	6	4	4
Quality improvement officers	6	4	6	3	3
Healthcare professionals	5	5	6	4	4
Authorities, health insurers, and other purchasers of healthcare	6	4	6	4	2
Patients/relatives of patients	-	4	4	-	-

This number presents the contribution all stakeholder groups made (of all mentioned strategies) to the elements of the intervention program.

All components of the tailored intervention program were derived from the many mentioned strategies during the group interviews. Researchers were the first group who took part at the group interviews, and they mentioned all the components that were incorporated into the intervention programs in three countries: Germany (6 out of 6), Norway (6 out of 6), and Poland (4 out of 4). The other stakeholders mentioned also some of the components in those countries. Not all the components of the intervention program were mentioned by the researchers in the Netherlands and the UK. In the Netherlands, the researchers mentioned strategies contributing to five out of the seven elements in the intervention program. The contribution of the other stakeholders resulted in one additional element in the program. The researchers of the UK team mentioned four of the five elements of the intervention program. The other stakeholders did not mention additional elements for the intervention program.

Additionally, we assessed the contribution of the patient groups. In the Netherlands, patients provided suggestions contributing to four of the seven elements of the intervention program. The relatives of patients in Norway mentioned suggestions contributing to four of the six intervention elements.

## Discussion

### Main findings

Group interviews with stakeholders provided many strategies for implementing evidence-based chronic illness care. The number of strategies varied more between countries than between groups within each of the countries. The highly productive groups seemed to be those of healthcare professionals and healthcare researchers, but this finding has to be interpreted carefully because the group of healthcare professionals consisted of two merged groups in three countries. The added value of structured interviews after brainstorming was highly variable, but in three countries, it led to substantially more strategies. All interviewed stakeholders mentioned strategies that were incorporated in the tailored intervention programs, which are subsequently tested. The type of strategies generated and their actual use in intervention programs generally did not differ systematically between stakeholder groups.

### Interpretation

Our study used brainstorming, which is based on the assumption that with increasing volume of strategies the number of “good strategies” will also increase [4]. The ultimate proof for this will be provided by the five trials [9-13], which examine the processes and outcomes of implementation programs that were based on the suggestions made in the group sessions. We felt that it is difficult to assess the “validity” of the strategies generated in the group interviews as we could not think of a meaningful reference for such assessment. Nevertheless, we believe that this exploratory study provides valuable insights that help to interpret results of group sessions to generate ideas, also because comparative research on group interview methods is limited.

Group interviews with healthcare professionals and patients have been successfully used in previous studies to help develop strategies to facilitate the implementation for mentioned barriers and enablers [16,17]. It was striking that the types of strategies of different stakeholders were overall similar if mapped out onto a pre-defined framework [6]. Other researchers found that stakeholder groups who were individually interviewed [18] or filled out a survey [19] did not differ in their perceptions, a finding that partly corresponds with this study. However, these studies were not using healthcare professionals. The number of strategies seemed to vary more between countries than between groups. These differences in the results can be due to country-specific reasons (“cultural”) or different healthcare systems [20] or due to the different chronic conditions (multi-morbidity, cardiovascular disease, depression in the elderly, COPD, and obesity) in each country.

### Strengths and weaknesses

This study gives extensive information about 25 group interviews in five countries, which is substantially more than in many other group interview studies [21]. The heterogeneity of chronic conditions and healthcare settings adds to the robustness of our findings, but it might also have biased the analyses in unpredictable ways. We did not check whether the study had identified all possible strategies (e.g., by doing more group interviews in each of the stakeholder groups in each of the countries), because this was not feasible. The written international study protocol contributed to the coherence of the study, but nevertheless, the procedures were executed in slightly different ways In particular, the Norwegian team did not manage to perform a structured phase, and in Poland, one group session could not be arranged and was replaced by three individual interviews. The small effect of group size and interview time on the number of strategies mentioned during brainstorming and structured interview needs to be examined in future studies. Interview group size did not have substantial effect in our study, while other research showed mixed effects [22,23]. Use of suggested strategies in the implementation programs was intended to be a proxy of usefulness, but use may in fact reflect various criteria: perceived effectiveness, feasibility, preference, or acceptability among the intervention design team.

### Recommendations for practice and research

Further studies of methods for tailoring interventions to determinants in healthcare are recommended to provide more insight, because this is to our knowledge the first comparative study on the topic. On the basis of the results, and of a qualitative content analysis [6], we suggest carefully considering which stakeholder groups to involve as we found few differences in the types of suggestions for improving healthcare practice. The groups of researchers provided nearly all components of the implementation programs, which could illustrate both their broad knowledge of how to improve healthcare practice, the setting, and their task and their rejection of specific suggestions made by other stakeholders. Involving stakeholders expected to contribute to the trustworthiness and impact of implementation programs and should be included in future studies. Future studies might consider a broader range of methods of involving stakeholders, such as electronic brainstorming sessions, e.g., interactive computer systems or using a phone-based application that supports *ad hoc* brainstorming sessions [24], conference meetings, or telephone meetings, because bringing groups together is time-consuming and is not always possible [25], and because these alternative methods could reduce the costs.

As this study is one of the first on the topic, we are careful with providing strong recommendations for practice. Our study suggests that an efficient approach to develop a tailored implementation program may be to start with a group interview with a productive group (clinicians or researchers), subsequently followed by interviews in other stakeholder groups until no new information is received. Involving various stakeholders in group interviews may have the (primary or additional) purpose to enhance the credibility of an implementation program. If this is the case, procedures and results may be less relevant in later interviews given the focus on buy-in of stakeholders.

## Conclusion

The five types of stakeholders mentioned many strategies for improving healthcare for patients with chronic diseases. Group size and interview time had no relevant effect on the number of strategies generated. Our study shows that the type of strategies did not vary between the stakeholders within the participating countries. With structured interviews involving a systematic assessment and presentation of given determinants of practice and results of research on interventions, discussion between the group participants are recommended if feasible, because these interviews provided a substantial number of additional strategies compared to the brainstorming phases. This implies that group interviews need to be carefully prepared in order to optimize their added value.

The strategies gathered from brainstorming and structured interviews were used as starting points for the tailored intervention programs which will be implemented and tested in the next phase of the TICD project.

## Additional file

Additional file 1:
**Outline of TICD WP3.** Matching implementation interventions to identified determinants of practice.

## Abbreviations

UK: United Kingdom
TICD: Tailored implementation in chronic disease
CVRM: cardiovascular risk management
COPD: chronic obstructive pulmonary disease
