# Supplementary material for: Stakeholders’ contributions to tailored implementation programs: an observational study of group interview methods
Source: Implement Sci. 2014 Dec 6;9:185. doi: 10.1186/s13012-014-0185-x (PMC4268850; doi:10.1186/s13012-014-0185-x)
Supplement: Additional file 1: — Outline of TICD WP3. Matching implementation interventions to identified determinants of practice. [file 13012_2014_185_MOESM1_ESM.doc]

**Outline of TICD WP3 - Matching implementation interventions to identified determinants of practice- an outline**

Version of 8 January 2012, Michel Wensing

**Introduction**

Work package 3 focuses on the logical next step in tailoring after identification of determinants of practice (“barriers and enablers of change”), which is: choosing and optimizing implementation interventions, so that they are optimally linked to determinants of practice. Work package 3 has been planned for months 19 to 30 in the TICD project (=August 2012 - July 2013; 12 months in total). Appendix 1 lists the activities, which have been specified in the TICD project application. Given the progress so far and the summer holiday, the actual start will indeed be in August 2012. Our intention is to complete work package 3 research earlier than planned, ideally December 2012. This would give us 24 months for the main study in WP4 (January 2013-December 2014). This document presents an outline for WP3.

| **Box 1. Simplified introduction to the concept of tailoring interventions to determinants**  A parallel can be made with patient care. After a diagnosis is made in a patient, a range of treatment options may be available. When planning a program to improve healthcare, this is exactly the same. Some factors may result logically in a specific type intervention (e.g. lack of knowledge implies that education is needed to solve this), for other factors a range of options is available. For instance: resistance to change may be tackled by providing convincing evidence to clinicians, by involving an opinion leader in a continuing education program, by financial incentives for desired behaviors, by going around specific clinicians by reallocation of tasks, etc.. Appendix 2 provides a widely used list of implementation interventions. Tailoring means that the type of intervention chosen is adapted to targeted individuals. Furthermore, like some treatments of patients have to be optimized (e.g. regarding dosing of medication), some implementation interventions also have to be optimized (e.g content of professional education). Tailoring also means that content or logistical specifics of interventions are adapted to targeted individuals. |
| --- |

**Concepts of tailoring**

The concept of “tailoring interventions to relevant determinants” is often used in a loose way, but it has in fact different dimensions. Historically, tailoring implementation interventions has not been well defined and conceptualized and the TICD project aims to contribute to such conceptual clarification Relevant features of tailoring include:

-intensity of tailoring: matching interventions to determinants at population-level (e.g. all participants in a national project), at practice-level (e.g. outreach visits to all practices to assess needs), and clinician-level (e.g. using some interviewing method). The term ‘intensity’ is taken from the Cochrane review on tailoring, but it could be debated whether other terms would be more appropriate (e.g. ‘level of tailoring’).

-timing of tailoring: matching interventions to determinants of change at the design stage of an improvement project (when planning interventions) and at the delivery stage of a project (when running the project).

-tailoring at the level of the intervention or within chosen interventions: choice of global type of implementation interventions (e.g. professional education, financial incentives or specific behavior change mechanism) versus optimization of chosen interventions (e.g. content of professional education, specifics and budget involved in financial incentives)

-tailoring rationale: Explicit reason given to chose the intervention as a component of the tailored program, such as perceived impact/importance (why the intervention might have an important effect) and feasibility/cost.

There may be other characteristics to classify matching methods.

**Methods currently used for tailoring interventions**

There is a range of methods available for tailoring, although not every method may be useful for each type of tailoring. Appendix 3 describes these tailoring methods in some more detail. These methods include:

- Open interview methods (individual or in groups) in which potential implementation interventions are identified and assessed, given the known determinants of practice;
- Structured interview methods, guided by checklists and research evidence, in which implementation interventions are linked to known determinants in some systematic way, using templates
- Discrete choice experiments, in which scenarios are systematically varied to assess the preferences for specific intervention components;
- Intervention modeling experiments, in which an variations of an implementation strategy is applied on individuals and self-reported outcomes are documented;
- Quantitative modeling, in which available data are used to identify characteristics of interventions that may be associated with better outcomes, usually in a regression analysis
- Developmental/action research, an approach where researchers follow the direct needs of knowledge users and the difference between research and action gets blurred.

**Aims of the study in TICD work package 3**

From literature reviews (WP1) we learned that there is little (if any) comparative research on methods and concepts for tailoring implementation interventions to determinants of practice. In the TICD project proposal, we indicated that two up to four specific methods will be selected for linking interventions to determinants of practice. The strongest evidence for the usefulness of approaches to matching interventions will be provided by rigorous evaluations of tailored implementation interventions. This will be done in TICD work package 4. Here, we describe a work package 3 will focus on intermediate outcomes in terms of suggested implementation interventions and time involved.

The **overall aim** of work package 3 is to assess the usefulness of approaches to group interviews for developing a program for implementing a set of recommendations for clinical practice.

Specific aims for the planned research are:

1. To compare the implementation programs, which have been developed by groups, between different types of participants: implementation researchers, quality improvement staff, clinicians, other stakeholders (and patients).
2. To assess the added value of a structured interview method for developing the implementation program compared to an open (unstructured) interview method with respect to the content of the implementation programs.
3. To document the experiences and time involved of participants in the group interviews.
4. To document whether and how findings of the group interviews have been used to chose or develop an implementation program, which is studied in TICD work package 4.

The content of implementation programs will be analyzed with respect to (a) number and diversity of implementation interventions, (b) knowledge content, (c) behavior change mechanisms involved, (d) tailoring rationale, (e) organizational details.

**Research plan**

**Study design**

A prospective observational study is planned with a number of comparisons. In each of the national studies, participating groups start with (a) the same given list of what is to be improved (e.g. which aspect of preventive screening or drug prescribing), which is the same as in work package 2 (b) the same given list of determinants of practice, which is a consolidated result of work package 2. The groups are all invited to develop an implementation program, which may comprise one or more interventions. Four groups should be composed, which are interviewed separately (a fifth group of patients is optional). Each interview has two phases: an open phase followed by a structured phase. Table 1 provides a schematic overview.

**Table 1. Study design**

|  | Phase 1: Open interview method | Phase 2: Structured interview method |
| --- | --- | --- |
| Group 1: Implementation researchers (including TICD ) (n=4 to 8) | A | B |
| Group 2: Quality improvement people (n=4 to 8) | C | D |
| Group 3: Stakeholders group 1, typically clinicians (n=4 to 8) | E | F |
| Group 4: Stakeholders group 2, others (n=4 to 8) | G | H |
| Group 5 (optional): Patients (n=4 to 8) | I | J |

For Aim 1 we compare:

1. A-C-E-G (-I)
2. B-D-F-H (-J)

For Aim 2 we compare:

1. A-B; C-D; E-F; G-H; (I-J)

**Sample**

In each country, a purposeful sample is composed. Four groups of 4-8 individuals each should be created (any individual should be in only one group):

- Group 1 comprises of implementation researchers, including members of the TICD team and other academics with relevant expertise.
- Group 2 comprise of quality improvement people, not involved in the TICD team (individuals who develop or coordinate continuing education and quality improvement for the targeted patients, professionals or healthcare sector).
- Group 3 comprises of a specific type of stakeholders, namely those most relevant for the implementation, typically clinicians in the targeted field (typically, physicians and nurses), possibly with a special interest in the topic.
- Group 4 comprises of representatives more external stakeholders, such as authorities, health insurers, patient representatives, etc. (excluding implementation researchers and quality improvement people).
- Group 5 (optional) comprises of a mix of patients (or their relatives), not representatives of patient organizations.

Groups should be relatively homogenous. For instance, if mixed groups of physicians and nurses do not work well, you have to organize a separate group for physicians and a separate one for nurses.

In principle, we propose one interview session per group. After the group sessions (most likely November 2012), the coordinator will make an inventory to examine the level of saturation.

**Group interview format**

While group interviews may give logistical challenges of getting people together, we aim to do these because (a) the group interaction facilitates the interview, (b) most research teams indicated that these are feasible, (c) using the same method across countries adds to the comparability of methods. If a specific group is very difficult to include in a group sessions, we pragmatically use individual interviews for those and group interviews for others (no ‘missions impossible’).

**Outline of the interview**

The content of the interviews will differ across the countries/disease specific focus. This outline focuses on the structure and type of questions, but needs to be tailored to the relevant clinical content. Each group is invited to develop an implementation/improvement program (comprising one or more implementation interventions). In other words, they are invited to help the TICD team to develop the implementation program that will be tested in the next phase. Try to avoid a focus on study designs, research methods or outcome measures.

Each interview starts with an open approach, which is then followed by a structured method (clearly separated by a break). Avoid long monologues, avoid jargon from research or implementation science (e.g. EPOC terminology) and avoid giving directions or preferences except as indicated in the structured approach. The exact flow of the interviews depend on content, the participants, cultural habits, etc.. Annex 5 provides the form that should be used to record the results of the interview (and to some extent, also structure it). The outline for all interviews in this TICD WP3 study is as follows:

1. General introduction (indicative: 15 minutes)

a. Present TICD study and aims of interview briefly.

b. Present chosen targets for improvement (and underlying practice guideline when relevant). A total of 3-8 specific targets is suggested (e.g. ‘reduce prescribing of antidepressants’ , ‘monitor renal function in heart diseases patients’ etc.)

c. Present some data on performance gaps and/or practice variation related to the chosen targets (that is, show that improvement of healthcare is possible and warranted). Note: NOT evidence on implementation interventions.

d. Give every participant annex 5A on paper to present a standardized list of determinants of practice for chosen targets for improvement. This has to be prepared on the basis of WP2 research and has to be the same for each interview session. In principle, determinants have to be presented separately for each chosen target for improvement; a maximum of 10 determinants per target is suggested (and an overall maximum of 30 determinants per interview). For instance, if you have chosen 3 targets for improvement with 5 determinants each, the participants have to reflect on 15 different factors. When determinants of practice overlap across different targets for improvement, you may combine the lists of targets in an efficient way. For instance, in the Netherlands we will use one list of 11 determinants for all 6 targets for improving cardiovascular care.

e. Present the context for improvement in terms of available time, staffing, resources in the national TICD project. While groups may consider long-term ideas, it is important that they also consider short-term possibilities (e.g. within the TICD project).

2. Open interview part (indicative: 30 minutes)

a. Ask about solutions/approaches to address the listed determinants (start with brainstorming). Spontaneous categorization or prioritization by interviewed people is fine, but should not be actively encouraged by the interviewer. The group may simply generate a list of interventions, they may build an integrated program with a mix of interventions, they may indicate that more research is required before interventions can be chosen, they may indicate that tailoring at individual level is required to decide what intervention is applied, etc. (or combinations of these approaches).

b. The interviewer checks and asks about major omissions regarding goals/determinants and, when present, suggests to reflect on these. It is crucial to the interviewer/group moderator does not provide direction or guidance at this stage.

c. Complete Annex 5B: a short description of the action plan in words chosen by the interviewed people. In reality, notes are made in the group session (possibly on a flip-over) which are elaborated by the research team later (after and outside the group meeting) by adding information from the audiotape and checking by participations. In the group meeting, the interviewer should encourage to be specific in terms of activities, organization, and planning. If there are diverging views on the plan, try to reach consensus or consider how these may be dealt with in the action plan (e.g. you may wish to sort out specific issues). No structured template is used in the interview (but we ask country teams to present a categorization of the result, see below under data-analysis).

3. Break

4.Structured interview part (indicative 30 minutes)

a. Present implementation strategies and research evidence related to their potential impact in the chosen clinical domain (Annex 5C). Each national team should review the international evidence in the targeted clinical domain to prepared this form.

b. If the list of determinants of practice is very long, please ask to prioritize and chose the most relevant determinants to target.

c. Complete the structured form as specific in Annex 5, section D.

d. Ask to make an action plan for implementation (or adapt/add to the plan generated in the first phase) as indicated in Annex 5E. If there are diverging views on the plan, explore whether ideas can be combined or whether further actions are needed to come to a final plan.

5.Closure (indicative: 15 minutes)

a. Summarize proposals for implementation interventions

b. Seek confirmation for the summary.

c. Ask participants to complete a few questions as specified on Annex 5F.

**Preparation of data**

After the group sessions have been completed, the research teams have the following tasks for each group:

1. Elaborate the text on forms B and E from Annex 5 on the basis of audiotape of the group session. If possible, have the text checked by participants (indicate when this has indeed be done). Please add clarifications where needed for international readers.
2. Complete as the research team form D in Annex 5 for the open part of each session (so that we can compare these with the form D completed by each group).
3. Translate the forms into English for international analysis. You may try Google translator for this, but please check the result.

The raw data of the research comprise of completed forms as specified in Annex 5 (one form for each group).

**Data-analysis**

Each national research team should use the group interviews to inform the choice of interventions for research in TICD work package 4. Each team is free to use the interviews for scientific papers, particularly if focused on how to improve healthcare for the targeted chronic condition. Please coordinate with the international study coordinator before sending papers to international journals. The international data-analysis is done centrally and led by the study coordinator (MW) who will involve at least one researcher from each national team. This international comparison will focus on the conceptual and methodological questions in this study:

1) **Type of implementation interventions**: broad type of intervention/activity proposed, using the EPOC list, the TICD checklist, and possibly other lists to characterize the interventions

2) **Tailoring rationale**: explicitly given reason choice of intervention/activity, to be classified into (a) impact/importance of determinant that is targeted, (b) perceived impact of the chosen implementation intervention, (c) research evidence underlying the effect of interventions, (d) feasibility and cost, (e) other considerations.

3**) Tailoring included in the program at its delivery**: whether and how further tailoring is included in the proposed approach, considering (a) intensity (population/practice/practitioner), (b) focus (exposure to interventions or design of intervention components), (c) methods used (interviews, questionnaires, etc.), (d) explicit rationale given for inclusion of tailoring

4) **Theoretical** b**ehavior change mechanisms** that are explicitly mentioned, if any (e.g. role modeling, price elasticity, leadership, etc.), using Theoretical Domains Framework (TDF), Normalisation Process Theory (NDT), and possibly other frameworks.

5) **Knowledge content** of educational interventions, considering (a) knowledge, (b) skills and (c) attitudes targeted. We will examine whether further post-hoc classification is knowledge content is possible.

6**) Practical details** of the action plans, considering (a) time planning, (b) support staff required, (c) resources required. Here we will seek a post-hoc classification, because it is difficult how much detail can be expected.

Other areas of interest may emerge during analysis. The specific variables will be elaborated and applied by the Nijmegen team on the completed annex 5 forms. The result will be fed back to the project teams for verification.

For research question 3, we simply document time and experiences of participants in the group interviews. For research question 4, we will use data from work package 4 .

**Scientific papers**

We plan at least a collaborative paper (across five studies) on the methods for tailoring in 2013. The work package coordinator will lead this paper, involving researchers from each national study team. In addition, each research team is encouraged to write a paper describing the content of findings. So, ideally we have 6 scientific papers from this work package 3.

**Annex 1. Text from project application on work package 3 (slightly adapted)**

**Step 1. Selection of matching methods and elaboration of the study protocol**

Planning: April-July 2012

We had originally planned to perform a Delphi-procedure in the TICD team to select up to four methods for matching implementation interventions to identified determinants of practice. Our experience in WP2 regarding the Delphi procedure was that discussion and qualitative comments were most informative, while the structured assessments did not discriminate well between methods. Therefore a revised approach was used, implying that we started with collecting comments and rating methods in the plenary session in April 2012 in Heidelberg. Then we used email and telephone contact to finalize the choice and refinement of the study protocol.

**Step 2. Standardization of determinants of practice.**

Planning: July-August 2012

The list of determinants of practice resulting from work package 2 will be standardized to set the stage for the next steps. Focus on the list of plausibly important determinants, or a subset of these if the list is long. This is required to create an equal starting point for the planned evaluation. Note that this will be done within each country/project; there is no intention of making a standardized list of determinants across all countries/projects. The determinants will be linked to specific recommendations for clinical practice, which we want to implement. However, if determinants largely overlap, you can combine the lists of determinants for different goals of improvement.

An issue is at what level of detail this will be done to avoid, for instance, that one project defines n < 10 key determinants and the other project n > 200 determinants. These may be similar or different across different recommendations. We suggest to identify not more than 10 determinants for each recommendation, and a maximum of 30 determinants in total (fewer if possible). If relevant, connections between the factors may be specified. E.g. some factors may be underlying to other factors. The result of this phase is a structured list/diagram of interconnected goals for improvement and determinants of practice, which provides input for the research planned in step 3.

**Step 3. Comparative evaluations.**

Planning: Data collection in August-December 2012, analysis and report: Jan – March 2013.

Then head-to-head comparisons of “methods” will be conducted for each of the targeted chronic conditions (i.e. discrete but related studies). This internationally standardized protocol for these studies guides these studies. Deviations are only possible if they do not disturb the procedures planned in this protocol. The comparisons are designed as comparative evaluations (randomized trials, or -more likely- prospective observational evaluations). We will take care that study groups cannot influence each other during the study (to avoid contamination), that “interventions” to match implementation interventions are well defined and implemented, and that measures for evaluation are standardized.

**Annex 2. Current EPOC of implementation interventions**

**A Professional interventions**

a) Distribution of educational materials (Distribution of published or printed recommendations for clinical care, including clinical practice guidelines, audio-visual materials and electronic publications. The materials may have been delivered personally or through mass mailings.)

b) Educational meetings (Health care providers who have participated in conferences, lectures, workshops or traineeships.)

c) Local consensus processes (Inclusion of participating providers in discussion to ensure that they agreed that the chosen clinical problem was important and the approach to managing the problem was appropriate.)

d) Educational outreach visits (Use of a trained person who met with providers in their practice settings to give information with the intent of changing the provider’s practice. The information given may have included feedback on the performance of the provider(s).

e) Local opinion leaders (Use of providers nominated by their colleagues as ‘educationally influential’. The investigators must have explicitly stated that their colleagues identified the opinion leaders.)

f) Patient mediated interventions (New clinical information (not previously available) collected directly from patients and given to the provider e.g. depression scores from an instrument.)

g) Audit and feedback (Any summary of clinical performance of health care over a specified period of time. The summary may also have included recommendations for clinical action. The information may have been obtained from medical records, computerised databases, or observations from patients.)

The following interventions are excluded:

• Provision of new clinical information not directly reflecting provider performance which was collected from patients e.g. scores on a depression instrument, abnormal test results. These interventions should be described as patient mediated.

• Feedback of individual patients’ health record information in an alternate format (e.g. computerised). These interventions should be described as organisational.

h) Reminders (Patient or encounter specific information, provided verbally, on paper or on a computer screen, which is designed or intended to prompt a health professional to recall information. This would usually be encountered through their general education; in the medical records or through interactions with peers, and so remind them to perform or avoid some action to aid individual patient care. Computer aided decision support and drugs dosage are included.)

i) Marketing (Use of personal interviewing, group discussion (‘focus groups’), or a survey of targeted providers to identify barriers to change and subsequent design of an intervention that addresses identified barriers.)

j) Mass media ((i) varied use of communication that reached great numbers of people including television, radio, newspapers, posters, leaflets, and booklets, alone or in conjunction with other interventions; (ii) targeted at the population level.)

k) Other (Other categories to be agreed in consultation with the EPOC editorial team.)

**B Financial interventions**

Provider interventions

a) Fee-for-service (provider has been paid for number and type of service delivered)

b) Prepaid (no other description)

c) Capitation (provider was paid a set amount per patient for providing specific care)

d) Provider salaried service (provider received basic salary for providing specific care)

e) Prospective payment (provider was paid a fixed amount for health care in advance)

f) Provider incentives (provider received direct or indirect financial reward or benefit for doing specific action)

g) Institution incentives (institution or group of providers received direct or indirect financial rewards or benefits for doing specific action)

h) Provider grant/allowance (provider received direct or indirect financial reward or benefit not tied to specific action)

i) Institution grant/allowance (institution or group of providers received direct or indirect financial reward or benefit not tied to specific action)

j) Provider penalty (provider received direct or indirect financial penalty for inappropriate behaviour)

k) Institution penalty (institution or group of providers received direct or indirect financial penalty for inappropriate behaviour)

l) Formulary (added or removed from reimbursable available products)

m) Other (other categories to be agreed in consultation with the EPOC editorial team)

Patient interventions

a) Premium (Patient payment for health insurance. It is important to determine if the patient paid the entire premium, or if the patient’s employer paid some of it. This includes different types of insurance plans.)

b) Co-payment (Patient payment at the time of health care delivery in addition to health insurance e.g. in many insurance plans that cover prescription medications the patient may pay 5 dollars per prescription, with the rest covered by insurance.)

c) User-fee (Patient payment at the time of health care delivery.)

d) Patient incentives (Patient received direct or indirect financial reward or benefit for doing or encouraging them to do specific action.)

e) Patient grant/allowance (Patient received direct or indirect financial reward or benefit not tied to specific action.)

f) Patient penalty (Patient received direct or indirect financial penalty for specified behaviour e.g. reimbursement limits on prescriptions.)

g) Other (other categories to be agreed in consultation with the EPOC editorial team)

**C Organisational interventions**

Provider orientated interventions

a) Revision of professional roles (Also known as ‘professional substitution’, ‘boundary encroachment’ and includes the shifting of roles among health professionals. For example, nurse midwives providing obstetrical care; pharmacists providing drug counselling that was formerly provided by nurses and physicians; nutritionists providing nursing care; physical therapists providing nursing care. Also includes expansion of role to include new tasks.)

b) Clinical multidisciplinary teams (creation of a new team of health professionals of different disciplines or additions of new members to the team who work together to care for patients)

c) Formal integration of services (bringing together of services across sectors or teams or the organisation of services to bring all services together at one time also sometimes called ‘seamless care’)

d) Skill mix changes (changes in numbers, types or qualifications of staff)

e) Continuity of care (including one or many episodes of care for inpatients or outpatients)

• Arrangements for follow-up.

• Case management (including co-ordination of assessment, treatment and arrangement for referrals)

f) Satisfaction of providers with the conditions of work and the material and psychic rewards (e.g. interventions to ‘boost morale’)

g) Communication and case discussion between distant health professionals (e.g. telephone links; telemedicine; there is a television/video link between specialist and remote nurse practitioners)

h) Other (other categories to be agreed in consultation with the EPOC editorial team)

Patient orientated interventions

a) Mail order pharmacies (e.g. compared to traditional pharmacies)

b) Presence and functioning of adequate mechanisms for dealing with patients’ suggestions and complaints

c) Consumer participation in governance of health care organisation

d) Other (other categories to be agreed in consultation with the EPOC editorial team)

Structural interventions

a) Changes to the setting/site of service delivery (e.g. moving a family planning service from a hospital to a school)

b) Changes in physical structure, facilities and equipment (e.g change of location of nursing stations, inclusion of equipment where technology in question is used in a wide range of problems and is not disease specific, for example an MRI scanner.)

c) Changes in medical records systems (e.g. changing from paper to computerised records, patient tracking systems)

d) Changes in scope and nature of benefits and services

e) Presence and organisation of quality monitoring mechanisms

f) Ownership, accreditation, and affiliation status of hospitals and other facilities

g) Staff organisation

h) Other (other categories to be agreed in consultation with the EPOC editorial team)

**D Regulatory interventions**

Any intervention that aims to change health services delivery or costs by regulation or law. (These interventions may overlap with organisational and financial interventions.)

a) Changes in medical liability

b) Management of patient complaints

c) Peer review

d) Licensure

e) Other (other categories to be agreed in consultation with the EPOC editorial team)

Reference

-Cochrane Effective Practice and Organisation of Care Group (EPOC). Data collection checklist. Ottawa: EPOC, 2002. Http://epoc.cochrane.org/sites/epoc.cochrane.org/ files/uploads/datacollectionchecklist.pdf

**Annex 3. Alternative list of implementation interventions**

Powell BJ, McMillen JC, Proctor EK, Carpenter CR, Griffey RT, Bunger AC, Glass JE, York LY. A compilation of strategies for implementing clinical innovations in health and mental health. Med Care Res Rev 2012;69:123-157.

**Abstract**

Efforts to identify, develop, refine, and test strategies to disseminate and implement

evidence-based treatments have been prioritized in order to improve the quality

of health and mental health care delivery. However, this task is complicated by an

implementation science literature characterized by inconsistent language use and

inadequate descriptions of implementation strategies. This article brings more depth

and clarity to implementation research and practice by presenting a consolidated

compilation of discrete implementation strategies, based on a review of 205

sources published between 1995 and 2011. The resulting compilation includes 68

implementation strategies and definitions, which are grouped according to six key

implementation processes: planning, educating, financing, restructuring, managing

quality, and attending to the policy context. This consolidated compilation can serve

as a reference to stakeholders who wish to implement clinical innovations in health

and mental health care and can facilitate the development of multifaceted, multilevel

implementation plans that are tailored to local contexts.

**Annex 4. Inventory of methods for matching interventions to barriers and enablers for change**

**Semi-structured interview methods**

These include individual interviews, brainstorming, and focus group interviews. These methods are widely used in practice, usually in combination with an effort to identify determinants of practice. Checklists or planning models are not used. This type of methods may provide a ‘reference group’, against other methods have to prove their value. Specific examples in research seem rare, but here is an example:

*Curran GM, Mukherjee S, Allee S, Owen RR. A process for developing an implementation intervention: QUERI series. Implem Sci 2008;3:17.*

**Methods guided by checklists (planning models)**

Any method can be structured according to a chosen checklist /framework for linking determinants of practice to implementation interventions. The format may vary from a single session to a series of steps with various types of activities. It is usually facilitated by researchers or support staff; the complex methods may include literature reviews and mapping exercises. There is a range of checklists and planning models so we would have to make a choice, if we would decide to include this type of methods in our research. Examples:

*Howat P, Jones S, Hall M, Cross D, Stevenson M. The precede-proceed model: application to planning a child pedestrian injury prevention program. Inj Prev 1997;3:282-287.*

*Brosseau LM, Parker DK, Lazovich D, Milton T, Dugan S. Designing intervention effectiveness studies for occupational health and safety: the Minnesota wood dust study. Am J Industr Med 2002;41:54-61. (uses PRECEDE-PROCEED)*

*Day L, Finch C, Hille KD et al. A protocol for evidence-based targeting and evaluation of statewide strategies for preventing falls among community-dwelling older people in Victoria, Australia. Inj Prev 2011;17:e3.*

*Heinen MM, Bartholomew LK, Wensing M, Van de Kerkhof P, Van Achterberg T. Supporting adherence and healthy lifestyles in leg ulcer patients: systematic development of the Lively Legs program for dermatology outpatient clinics. Pat Educ Counsel 2006;61:279-291.*

*Schmid AA, Andersen J, Kent T, Williams LS, Damush TM. Using intervention mapping to develop and adapt a secondary stroke prevention program in Veterans Health Administration medical centers. Implemen Sci 2010;5:97.*

*Van Bokhoven MA, Kok G, Van der Weijden T. Designing a quality improvement intervention: a systematic approach. Qual Saf Health Care 2003;12: 215-220.*

**Intervention modeling experiments**

Intervention modeling is a proposed step in the development of complex interventions. Modeling experiments aim to test one or more selected interventions in a simulated situation, using written scenarios and/or self-report measures of performance. These studies may be most useful in later stages of the intervention development, for making a final choice of intervention components. Modeling use real health professionals, but simulated measures (e.g. self-report questionnaires) and potentially also a subset of all intervention components. Examples:

*Bonetti D, Eccles M, Johnston M, Steen N, Grimshaw J, Baker R, Walker A, Pittls N. Guiding the design and selection of interventions to influence the implementation of evidence-based practice: an experimental simulation of a complex intervention trial. Soc Sci Med 2005;60:2135-2147.*

*Treweek S, Ricketts IW, Francis J, Eccles M, Bonetti D, Pitts NB, MacLennan G, Sullivan F, Jones F, Weal M, Barnett K. Developing and evaluating interventions to reduce inappropriate prescribing by general practitioners of antiobiotics for upper respiratory tract infections: A randomized controlled trial to compare paper-based and web-based modeling experiments. Implem Sci 2011;6:16.*

*Hrisos S, Eccles M, Johnston M, Francis J, Kaner E, Steen N, Grimshaw J. An intervention modelling experiment to change GPs' intentions to implement evidence-based practice: Using theory-based interventions to promote GP management of upper respiratory tract infection without prescribing antibiotics. BMC Health Services Research 2008: 8;10.*

*Hrisos S, Eccles M, Johnston M, Francis J, Kaner E, Steen N, Grimshaw J. Developing the content of two behavioural interventions. Using theory-based interventions to promote GP management of upper respiratory tract infection without prescribing antibiotics. BMC Health Services Research 2008: 8;11*

*Foy R, Francis JJ, Johnston M, Eccles M, Lecouturier J, Bamford C, Grimshaw J. The development of a theory-based intervention to promote appropriate disclosure of a diagnosis of dementia. BMC Health Services Research 2007, 7:207.*

*Lecouturier J, Bamford C, Hughes JC, Francis JJ, Foy R, Johnston M Eccles MP. Appropriate disclosure of a diagnosis of dementia: identifying the key behaviours of 'best practice'. BMC Health Services Research 2008,* ***8****:95.*

**Discrete choice experiments**

A structured and quantitative method would be a discrete choice experiment, although the one experience I know was not very positive (very low response rate). Example:

*Van Helvoort-Postulart D, van der Weijden T, Dellaert BG, de Kok M, von Meyenfeldt MF, Dirksen CD. Investigating the complementary value of discrete choice experiments for the evaluation of barriers and facilitators in implementation research: a questionnaire survey. 1. Implement Sci. 2009 Mar 1;4:10.*

**Quantitative modeling using practice variation**

Some implementation interventions, such as organizational changes and changes in the financial incentives, are difficult to apply and test in a controlled study. An alternative might to model the impact of such changes, using real data on practice variation. These data may be derived from published research or available databases. Application of the method requires quantitative skills, e.g. to run markov models. In the example below, we modeled the impact of a major change in referring patterns on health outcomes and costs.

*Harmsen M, Adang E, Wolters RJ, Van der Wouden JC, Grol R, Wensing M. Prevention of renal failure in children with urinary tract infections: a literature review and economic analysis. Value Health 2008;12:466-472.*

**Developmental / action research**

Researchers may work closely with participants to meet practical goals. There is a continuing cycle of research and action, so that the boundaries between the two gets blurred. Many of the so called ‘quality improvement projects’ may fit in this approach. Example:

*Bhutta A, Gilliam C, Honeycutt M, et al. Reduction of bloodstream infections associated with catheters in paediatric intensive care unit: stepwise approach. BMJ 2007;334:362-5.*

**Annex 5. Form for the planned group interviews**

**5A. Consolidated list of determinants of practice**

*Prepared by the national research team before the interview starts. A maximum of 3-8 targets for improvement is proposed and a maximum of 10 determinants per target/ 30 determinants in total in each interview.*

**Goal/target for improvement**: *describe one more goals/targets*

**Main determinants of practice, related to this goal/these goals:**

1 xxx

2 xxx

3 xxx

4 xxx

5 xxx

6 xxx

7 xxx

8 xxx

9 xxx

10 xxx

**5B. RESULT OF OPEN INTERVIEW: PROPOSED INTERVENTIONS/ACTIVITIES AND ACTION PLAN**

| *Text in words of the participants.(make notes during the group session, readable text after the session)* |
| --- |

**5C. PRESENTATION OF RESEARCH EVIDENCE ON IMPLEMENTATION INTERVENTIONS**

*Please present a brief review of research evidence on implementation/improvement interventions in the targeted clinical area (e.g. interventions for improving healthcare for patients with COPD). You may use powerpoint and/or a written table for this. Use a structured format for presenting the evidence, for instance the categories suggested below. When possible, you may present the interventions in relation to the specific goals for improvement and/or determinants of practice (a template linked to the TICD checklist will be provided by WP1 team - Andy Oxman). It is also suggested to include a generic summary of research evidence on improving chronic illness care, such as the proposed text below.*

***Format A***

a. Interventions targeted at health professionals

(e.g. professional education, audit and feedback, decision support)

b. Interventions targeted at patient care teams and practice organization

(e.g. revision of professional roles, introduction of case managers)

c. Interventions targeted at patients and carers

(e.g. implementation of decision aids or self-management programs)

d. Interventions targeted at organization of healthcare services

(e.g. change of leadership, merging of organizations, interventions on culture)

e. Interventions targeted at financial and other structures

(e.g. pay for performance, change of laws and regulations)

f. Complex interventions to improve healthcare

(combinations of types of interventions)

**Format B**

See page 12 or Powell BJ, McMillen JC, Proctor EK, Carpenter CR, Griffey RT, Bunger AC, Glass JE, York LY. A compilation of strategies for implementing clinical innovations in health and mental health. Med Care Res Rev 2012;69:123-157.

**Proposed generic summary of evidence**

A review of 142 randomized trials on improving diabetes care found that various improvement interventions improved glycated haemoglobin (-0.37 difference), LDL cholesterol (-0.10 mmol), systolic blood pressure (-3.1 mm Hg) and diastolic blood pressure (-1.5 mm Hg). A range of improvement intervention had positive effects, with financial incentives and continuous quality improvements as exceptions (no effects found). Interventions with highest effects appeared to be promotion of self-management, case management, and team changes. Larger effects were found when baseline values indicated poorer health status. Various aspects of professional practice (e.g. use aspirins and statins) also improved as a result of improvement interventions. The generalizability of these findings across settings and other conditions should be considered.

Trico AC et al. Effectiveness of quality improvement strategies on the management of diabetes: a systematic review and meta-analysis. Lancet 2012. DOI: 1016/S01406736(12)60480-2.

**5D. TEMPLATE FOR THE STRUCTURED INTERVIEW**

*Please ask the group to complete the form below for each goal for improvement.* *(simplified worksheet 4 from TICD WP1)*

**Goal/target for improvement**: *describe one more goals*

*Please specify one determinant and one intervention per row.*

| **Determinant prioritized by the group** (use numbers or short labels from Annex 5A) | **Intervention proposed by the group** (describe briefly) | **Why is this intervention suggested?** (see categories below *) | **Should the intervention be tailored to individuals or practices?** (yes/no) | **If tailoring to individuals/practices is recommended, please describe how this is done.** |
| --- | --- | --- | --- | --- |
|  |  |  |  |  |
|  |  |  |  |  |
|  |  |  |  |  |
|  |  |  |  |  |
|  |  |  |  |  |
|  |  |  |  |  |
|  |  |  |  |  |
|  |  |  |  |  |
|  |  |  |  |  |
|  |  |  |  |  |
|  |  |  |  |  |

* Reasons for selecting this implementation/improvement intervention or activity:

(1) impact/importance of determinant that is targeted

(2) perceived impact of the chosen implementation intervention

(3) research evidence underlying the effect of interventions

(4) high feasibility and low cost

(5) other considerations.

**5E. RESULT OF STRUCTURED PHASE: PROPOSED INTERVENTIONS/ACTIVITIES AND ACTION PLAN**

*Alternatively, you may note here only the changes and additions compared the result of the open phase.*

| Text in words of the participants. |
| --- |

**5F. RECORD OF PARTICIPANTS’ TIME AND EXPERIENCES**

*Make a short structured questionnaire in your language to record the following items for each session and participant.*

For researchers:

1. How much time was needed to prepare the interviews? (an estimation by the research team)
2. How long did the interview session take? (recorded for each session by interviewer)
3. How do you assess the feasibility of the chosen approach? (open question)

Please prepare a short structured from for each participant:

1. How much time did you need for preparation and travelling to the meeting? (total number of minutes)
2. Did you find the experience useful? (yes/partly/no/ don’t know) Please explain (open question)
3. Did you find the experience satisfactory? (yes/partly/no/ don’t know) Please explain (open question)
4. What would you do the same and what differently? (open question)

Please take care that you can record the findings in frequency tables and thematic summaries of questions to open questions.
